# Clinical Use of Probiotics in Pediatric Allergy (cuppa): A World Allergy Organization Position Paper

**DOI:** 10.1097/WOX.0b013e3182784ee0

**Published:** 2012-11-15

**Authors:** Alessandro Fiocchi, Wesley Burks, Sami L Bahna, Leonard Bielory, Robert J Boyle, Renata Cocco, Sten Dreborg, Richard Goodman, Mikael Kuitunen, Tari Haahtela, Ralf G Heine, Gideon Lack, David A Osborn, Hugh Sampson, Gerald W Tannock, Bee Wah Lee

**Affiliations:** 1 Department of Pediatrics - Division of Allergy - Pediatric Hospital Bambino Gesù - Rome, Vatican City; 2 Department of Pediatrics, University of North Carolina, Chapel Hill, NC, USA; 3 Department of Pediatrics and Medicine, Section of Allergy and Immunology, Louisiana State University Health Sciences Center, Shreveport, LA; 4 Department of Medicine, University of Medicine and Dentistry of New Jersey Medical School, Newark, NJ; 5 Department of Paediatrics, Imperial College London, London, UK; 6 Division of Allergy, Clinical Immunology and Rheumatology, Department of Pediatrics, Federal University of São Paulo, São Paulo, Brazil; 7 Department of Pediatric Allergology, Women's and Children's Health, University of Uppsala, Uppsala, Sweden; 8 Department of Food Science & Technology University of Nebraska, Lincoln, NE, USA; 9 Skin and Allergy Hospital, University of Helsinki, Helsinki, Finland; 10 Department of Allergy and Immunology, Royal Children's Hospital, University of Melbourne, Murdoch Childrens Research Institute, Melbourne, Australia; 11 King's College London, Asthma-UK Centre in Allergic Mechanisms of Asthma, Department of Paediatric Allergy, St Thomas' Hospital, London, UK; 12 Sydney Medical School, University of Sydney, New South Wales, Australia; 13 Jaffe Food Allergy Institute, Mount Sinai School of Medicine, New York, NY; 14 Department of Microbiology and Immunology, University of Otago, Dunedin, New Zealand; 15 Department of Paediatrics, National University of Singapore, Singapore

**Keywords:** probiotics, prevention of allergy, pediatric allergy

## Abstract

**Background:**

Probiotic administration has been proposed for the prevention and treatment of specific allergic manifestations such as eczema, rhinitis, gastrointestinal allergy, food allergy, and asthma. However, published statements and scientific opinions disagree about the clinical usefulness.

**Objective:**

A World Allergy Organization Special Committee on Food Allergy and Nutrition review of the evidence regarding the use of probiotics for the prevention and treatment of allergy.

**Methods:**

A qualitative and narrative review of the literature on probiotic treatment of allergic disease was carried out to address the diversity and variable quality of relevant studies. This variability precluded systematization, and an expert panel group discussion method was used to evaluate the literature. In the absence of systematic reviews of treatment, meta-analyses of prevention studies were used to provide data in support of probiotic applications.

**Results:**

Despite the plethora of literature, probiotic research is still in its infancy. There is a need for basic microbiology research on the resident human microbiota. Mechanistic studies from biology, immunology, and genetics are needed before we can claim to harness the potential of immune modulatory effects of microbiota. Meanwhile, clinicians must take a step back and try to link disease state with alterations of the microbiota through well-controlled long-term studies to identify clinical indications.

**Conclusions:**

Probiotics do not have an established role in the prevention or treatment of allergy. No single probiotic supplement or class of supplements has been demonstrated to efficiently influence the course of any allergic manifestation or long-term disease or to be sufficient to do so. Further epidemiologic, immunologic, microbiologic, genetic, and clinical studies are necessary to determine whether probiotic supplements will be useful in preventing allergy. Until then, supplementation with probiotics remains empirical in allergy medicine. In the future, basic research should focus on homoeostatic studies, and clinical research should focus on preventive medicine applications, not only in allergy. Collaborations between allergo-immunologists and microbiologists in basic research and a multidisciplinary approach in clinical research are likely to be the most fruitful.

## Ratification by Voting Member Societies of the World Allergy Organization October 2012

Albanian Society of Allergology and Clinical Immunology

American Academy of Allergy, Asthma and Immunology

American College of Allergy, Asthma and Immunology

Argentine Association of Allergy and Clinical Immunology

Argentine Society of Allergy and Immunopathology

Australasian Society of Clinical Immunology and Allergy

Austrian Society of Allergology and Immunology

Azerbaijan Society for Asthma, Allergy and Clinical Immunology

Brazilian Society of Allergy and Immunopathology

British Society for Allergy and Clinical Immunology

Bulgarian National Society of Allergology

Canadian Society of Allergy and Clinical Immunology

Colombian Allergy, Asthma, and Immunology Association

Croatian Society of Allergology and Clinical Immunology

Cuban Society of Allergology

Czech Society of Allergology and Clinical Immunology

Danish Society for Allergology

Dutch Society of Allergology

Egyptian Society of Allergy and Clinical Immunology

Egyptian Society of Pediatric Allergy and Immunology

Finnish Society of Allergology and Clinical Immunology

German Society for Allergology and Clinical Immunology

Honduran Society of Allergy and Clinical Immunology

Hong Kong Institute of Allergy

Hungarian Society of Allergology and Clinical Immunology

Icelandic Society of Allergy and Immunology

Indian College of Allergy, Asthma and Applied Immunology

Indonesian Society for Allergy and Immunology

Israel Association of Allergy and Clinical Immunology

Italian Society for Allergology and Clinical Immunology

Japanese Society of Allergology

Jordanian Society for Allergy and Clinical Immunology

Korean Academy of Allergy, Asthma and Clinical Immunology

Kuwait Society of Allergy and Clinical Immunology

Latvian Association of Allergists

Lebanese Society of Allergy and Immunology

Malaysian Society of Allergy and Immunology

Mexican College of Pediatricians Specialized in Allergy and Clinical Immunology Mongolian Society of Allergology

Norwegian Society of Allergology and Immunopathology

Panamanian Association of Allergology and Clinical Immunology

Philippine Society of Allergy, Asthma and Immunology

Polish Society of Allergology

Romanian Society of Allergology and Clinical Immunology

Russian Association of Allergology and Clinical Immunology

(Singapore) Allergy and Clinical Immunology Society

Slovenian Association for Allergology and Clinical Immunology

(South Africa) Allergy Society of South Africa

Spanish Society of Allergology and Clinical Immunology

(Sri Lanka) Allergy & Immunology Society of Sri Lanka

Swiss Society of Allergology and Immunology

Turkish National Society of Allergy and Clinical Immunology

(Thailand) Allergy, Asthma and Immunology Society of Thailand

Uruguayan Society of Allergology

### Contributing Regional Member Societies

American Academy of Allergy, Asthma and Immunology

American College of Allergy, Asthma and Immunology

Asia Pacific Association of Allergy, Asthma and Clinical Immunology

European Academy of Allergy and Clinical Immunology

Latin American Society of Allergy and Immunology

## Introduction

Humans have evolved to establish and maintain relative equilibrium with the dynamic and evolving microbial world. The body, inside and out, is cloaked with microbes that are likelier to be friends than enemies. Hence, the epidermal surface, respiratory tract, vagina, and large bowel are homes to biodiverse microbial communities (microbiota) containing mostly nonpathogenic bacterial species [1-3]. These bacteria are commonly referred to as commensals or symbionts because they form long-lasting interactive associations with their hosts. Inside the womb, the fetus lives in a sterile environment. However, during and immediately after birth, body surfaces, including the gastrointestinal tract, are adventitiously inoculated with bacteria of environmental and maternal origin. A regulated and predictable succession of bacterial groups is soon established. Those of the large-bowel microbiota are especially well studied [4-6]; less is known about the microbiota of the small intestine, airway, and skin. Alone among mammalian species, the large-bowel bacterial community of human infants is dominated during the first months of life by members of the genus *Bifidobacterium*. These bacteria are particularly capable of growing on milk oligosaccharides. The composition of the gut microbiota becomes more complex as the milk diet is supplemented with solid food and changes markedly after cessation of breastfeeding [5]. During early life, while immunological germinal foci develop in the bowel mucosa, the bowels of infants undergo a succession of changes with respect to the composition of the microbiota. Looked at immunologically, this succession must also represent a changing antigenic load of undeterminable size associated with the mass of bacterial organisms. Although most knowledge of the human microbiota is derived from studies of the bowel, similar changes probably occur in other parts of the body colonized by commensals. These changes may be as substantial as events in the bowel in forming the possible relationship between microbial exposure and allergies (see Establishing the Link Between Probiotic Administration and Disease Treatment). Immune homeostasis in the gut, sometimes called immune privilege, develops as a relationship is established between the intestinal microbiota, luminal antigens, and the epithelial barrier [7]. Tolerance to food antigens seems to require such a consortial relationship. Allergy may develop due to a failure to establish immune tolerance to nonharmful dietary proteins or to a loss of balance between immune suppression and sensitization. Many human genes are known to play some role in susceptibility to allergy development, but many environmental factors have also been implicated. As clinicians and scientists study whether interventions, such as the introduction of probiotic bacterial species, may be used to either initiate tolerance or restore a balance of tolerance, the complexity of the task is becoming evident. It seems essential to consider how each compartment of the body is affected by different microorganisms, what stage in the disease process is appropriate for intervention, and what dose of microorganism should be applied and for how long [8-10].

### 

#### 

##### Take-home message

• There are probably hundreds of explanations for the apparent surge in allergic disease in affluent countries over the past 60 years [11-14].

• Ethnicity and maternal diet, different hygiene standards and obstetric practices, and the frequency of use of antibiotics may account for some of the observed differences.

• Changes in lifestyle with a marked impact on exposure to bacteria and differences in bowel microbiota of infants born in countries with low or high prevalence of allergies have been most frequently cited [15,16].

• However, differences in patterns and abundance of microbes and parasitic worms between today and 60 years ago are not testable.

• The connection between the composition of the microbiota in early life, the programming of the immune system in terms of later response to environmental allergens, and predisposition of the child to allergies are the research topics we should focus on [17-21].

## Definitions and Objectives

For the purposes of this document, the following definitions will be used:

• Probiotics: microorganisms that "confer a health benefit on the host."[22] Hereafter, they will be defined as proprietary formulations of specific microorganisms (genus, species, and strain) and quantified populations of live bacteria that can be legally prescribed by physicians.

• Treatment: intervention targeting primary, secondary, or tertiary prevention; temporary relief; or cure.

• Supplementation: intervention targeting add-on, or adjuvant, therapy aimed at interfering with allergic mechanisms or homoeostatic processes, for efficient and sufficient treatment (as defined above).

• Microbiota: a bacterial community inhabiting a particular body site

• Commensals (or symbionts): the members of the microbiota.

• Metagenome: the collective genomes of the microbiota (sometimes this is alluded to as "microbiome," an equivocal term that will not be used in this document).

The aim of the present document was to examine the claims that probiotic supplements can be used for the treatment of allergic disease according to the above definitions.

## Epistemology

### The Translation From Prevention to Treatment

Probiotics, originally conceived for treatment of gastrointestinal diseases and later applied to allergy prevention, are increasingly proposed for the treatment of allergic disease [23-26]. This is a matter of concern for allergists because, despite the extensive literature on probiotics, the translation from possible prevention effects to therapeutic efficacy remains to be demonstrated. At issue are the limited number and quality of clinical trials and their reproducibility, [27] and several meta-analyses have refrained from recommending probiotics as therapeutic agents. This situation is likely to continue as long as appropriate indications, patient selection, strain-specific effects, doses, mechanisms of action, optimal initiation and duration of treatment, adequate follow-ups, product reliability, and availability have not been clearly established outside research settings.

### The Basic Assumption Behind Treatment is Based on Two Working Hypotheses

The hygiene hypothesis and the microbial origin of allergic disease hypothesis (see Hygiene Hypothesis, Microbial Origin of Allergic Disease Hypothesis, and Probiotics) have both been proposed to explain the epidemic of allergic disease. However, the question is whether there is sufficient proof from basic research to support or refute claims of clinical efficacy of specific probiotic interventions to modify the individual host response. No clear "biological conductor" or mechanism orchestrating the immunomodulatory effects necessary to establish tolerance that would allow a precise identification of specific probiotics that could overcome the allergy response has emerged. Additional basic and applied research is needed to develop avenues of treatment leading to evidence-based clinical applications.

### The Superorganism and the Implications of Microbiota Research

Clinicians are just beginning to realize the significance of a paradigm shift in biology that has occurred over the past 15 years. Our genetic landscape is infinitely more complex than hitherto imagined and must be viewed as an ecotope: human and microbial genomes assemble to interact in an evolutionarily conserved superorganism in which bacterial metagenomes and host metabolic networks cooperate toward the homoeostasis of the consortium [28,29]. Sophisticated statistical techniques (bioinformatics) are required to store, manage, and analyze the enormous amount of information on multilevel interrelationships (metadata) awaiting biological, physiological, and ecological interpretation and application [30]. The Human Microbiome Project, an international interdisciplinary effort to "generate resources enabling comprehensive characterization of the human microbiota and analysis of its role in human health and disease," highlights the many levels of complexity of this cutting-edge science [31]. As far as clinical applications are concerned, however, researchers are cautious in claiming mechanistic insights [32].

#### 

##### Take-home message

• The assumption that efficacy of supplementation in prevention implies efficacy in therapeutic applications is not borne out by clinical trial evidence.

• Current clinical science has not identified agents that are able to modify host disease phenotype, let alone individual host response.

• In the context of the superorganism, novel avenues of research are required before we can claim to have worked out the mechanisms of disease and treatment with oral supplementation.

## Allergy Terminology

This review aims to evaluate the possible clinical benefits of probiotics allergic diseases. For a meaningful discussion of the effects of supplementation interventions, it is necessary to define the endpoints to be evaluated, that is, allergy, atopy, sensitization, and allergic/atopic diseases.

### Allergy

In 2001, a task force within the European Academy of Allergology and Clinical Immunology (EAACI) proposed a new nomenclature for allergy, [33] and in 2004 a committee of the World Allergy Organization (WAO) issued a consensus statement on a global allergy nomenclature [34]. Only minor changes were made between 2001 and 2004; most importantly, the misleading term "atopic dermatitis" was replaced by "eczema."

**Figure 1:**
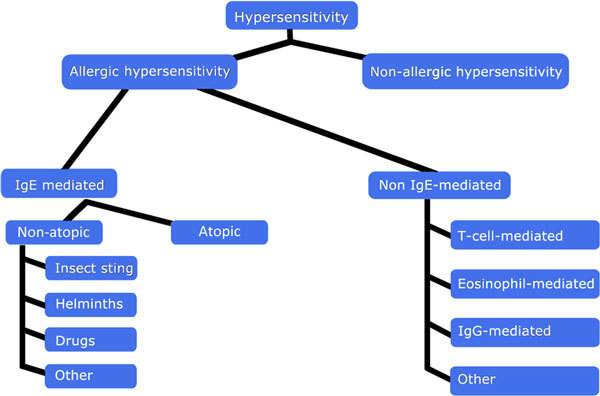
**A general outline of the subgroups of hypersensitivity **[34].

The basis of the global nomenclature is "hypersensitivity," that is, a reaction to a substance tolerated by "normal individuals." Hypersensitivity is divided into "nonallergic hypersensitivity," in cases when the immune system is not involved, and "allergic hypersensitivity" or "allergy" when reactions occur that involve the immune system or when such involvement is strongly suspected (Figure 1). Nonallergic hypersensitivity reactions are not within the scope of this article. Allergy can be divided into "immunoglobulin E (IgE)-mediated allergy" and "non-IgE-mediated allergy." Non-IgE-mediated allergic reactions can be mediated via, for example, IgG antibodies, T cells, and even by complement activation, that is, by Gell and Coombs type II, III and IV mechanisms (Figure 1) [9,10,35]. IgE-mediated allergy can be further divided into "atopic allergy," induced by allergen-specific IgE antibodies after exposure to minute amounts (picograms to nanograms) of antigenic molecules, and "nonatopic type of IgE-mediated allergy," which is characterized by IgE production after exposure to high amounts of allergen (micrograms to milligrams), often bypassing the cutaneous barrier (eg, Hymenoptera venoms or drugs) or after infestation by helminths (Figure 1). Nonatopic IgE-mediated allergy frequently occurs upon repeated exposure.

### Atopy

Atopy is defined by a World Allergy Organization (WAO)/EAACI document as "... a personal or familial tendency, usually in childhood or adolescence, to become sensitized and produce IgE antibodies in response to ordinary exposure to allergens, usually proteins. As a consequence, these persons can develop typical symptoms of asthma, rhinoconjunctivitis, or eczema." However, not all cases of eczema, asthma, and rhinoconjunctivitis are due to IgE mechanisms.

When discussing prevention and treatment of allergic sensitization or IgE-mediated allergic disease in infants, the definition may be simplified to: "Atopy is a tendency in the infant to become sensitized and produce IgE-antibodies in response to common allergens, sometimes expressed by developing symptoms such as asthma, rhinoconjunctivitis, or eczema."

Atopic sensitization, or the production of allergen-specific IgE antibodies, can be documented by in vitro IgE tests and in vivo tests, most often skin prick tests. However, different commercial brands of in vitro tests for IgE determination do not have the same sensitivity and specificity. Commercial tests have also increased their sensitivity over time. In some cases the allergen source materials bound to the solid phase have been changed, as have the anti-IgE antibodies for IgE detection. Skin prick tests, however, may yield different results depending on the technique used and the potency and composition of the allergen extracts. Thus, these methods must be defined in context. For comparison of sensitization between laboratories and within laboratories over time, the only reliable method is to analyze all samples by in vitro IgE tests using the same brand and batch of antigen. Comparison of skin test results are more accurately made when the same extracts from the same company are used, although the technique of the clinician is harder to standardize. Comparisons cannot be made between studies that use different methods for the estimation of allergen-specific sensitization.

### Allergic Diseases

**Figure 2:**
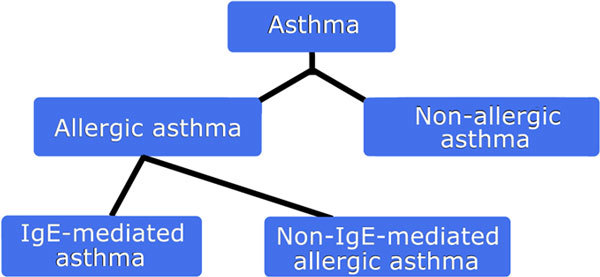
**The nomenclature of asthma **[34,35].

**Figure 3:**
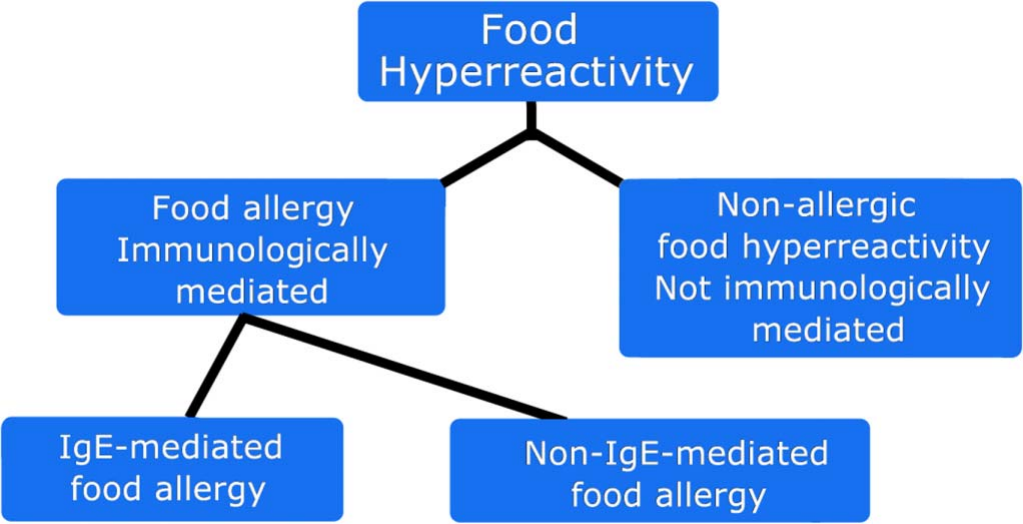
**The nomenclature of food allergy**.

**Figure 4:**
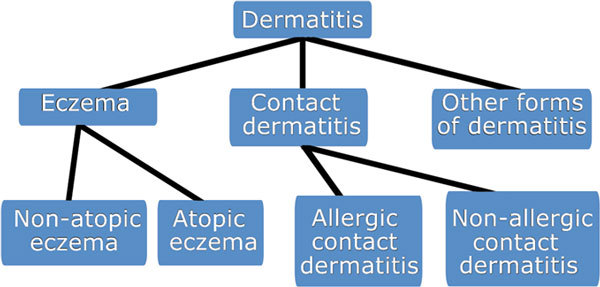
**The nomenclature of dermatitis and its immunologically mediated forms eczema (immunologically mediated dermatitis), IgE-mediated eczema (atopic eczema), and non-IgE-mediated eczema (nonatopic eczema) **[35].

The same principle that applies to the allergy nomenclature may be applied to allergic diseases. Thus, "allergic asthma" (ie, asthma with a probable or proven immune mechanism) can be divided into "IgE-mediated asthma" and "non-IgE-mediated asthma" (Figure 2). The latter, with cell involvement, should be distinguished from "nonallergic asthma," which refers to asthmatic conditions without involvement of the immune system (if they exist). Similarly, food allergy should be divided into "IgE-mediated food allergy" and "non-IgE-mediated food allergy" (Figure 3), and eczema should be divided as in Figure 4 These diseases themselves should derive their definitions from international documents such as the Global Initiative for Asthma [36] and Allergic Rhinitis and its Impact on Asthma guidelines [37]. However, it must be stressed that each of these phenotypic expressions of disease may have several mechanisms which, in turn, are likely to correspond at least in part to different genotypes. Thus, a prerequisite for the evaluation of prevention and treatment trials is to ensure correct use of the nomenclature to properly reflect the underlying effector mechanisms.

#### 

##### Take-home message

• In the evaluation of probiotic effect on allergic disease, the use of a correct terminology is of utmost importance.

• The effects of probiotics can be different in IgE-versus non-IgE-mediated allergic diseases.

• The possible effect of probiotics may be postulated to result from either direct immune mechanisms at the level of the T cell, B cell, or antigen-presenting cell for both IgE-mediated or non-IgE-mediated allergy (IgG mediated or T-cell mediated)

• Another series of mechanisms is possibly related to the shock organ, via lymphocyte or mast cell homing mechanisms, reduction in sensitivity of mast cells or basophils, or perhaps neural or smooth muscle cells associated with the skin or mucus membranes.

## Hygiene hypothesis, microbial origin of allergic disease hypothesis, and probiotics

Allergy is not a single disease, and multiple pathways are likely involved in both tolerance and sensitization. This explains why gene and disease linkages are so difficult to pinpoint. Yet, those studies that have found associations between gene alleles and allergic disease are limited in their findings, possibly due to incomplete definitions of disease and health, limitations of single-nucleotide polymorphism selection, or inappropriate segregation and interpretation [38,39].

The "hygiene hypothesis" proposes that, as a result of modern public health practices, individuals experiencing a relative deficiency in immune stimulation by microbes become vulnerable to the development of allergic hypersensitivities and their associated diseases [40]. The hypothesis was expanded when it became clear from studies of transgenic germ-free animals that the disruption or absence of gut microbiota accounts for the development of allergic airway responses in the absence of prior systemic priming [41]. As an extension of the hygiene hypothesis, the "microflora hypothesis of allergic disease" was postulated to highlight the role of the gut in modulating host immunity in early life [42] and possibly in later life [43]. Cross-sectional and cohort studies in young children with allergic diseases have shown an association between microbial patterns of colonization and allergic disease not displayed by healthy controls [44]. Additionally, an altered pattern of microbiota composition in early life, resulting in delayed colonization associated with the caesarean mode of delivery, has been associated with an increased risk of allergic disease [6]. High turnover of *Escherichia coli *strains in Pakistani children compared with Swedish children during the first 6 months after birth leads to stronger activation of the intestinal immune system [45]. Translocation of bacteria also occurs during the initial encounter; however, once an IgA-specific response to that strain of bacteria has developed, translocation and immune stimulation wanes [46]. Thus, supplementing with one probiotic strain might not be effective in modulating the immune response, and consideration should be given to supplementing with repeatedly changing probiotic strains in early life to maximize the stimulation and maturation of the immune system.

### 

#### 

##### Take-home message

• Genetics point to a multiple pathway etiology of allergic disease that has been marginally characterized by current research.

• The hygiene hypothesis points to individual susceptibility in certain host-environmental conditions but provides no quick fixes in either compartment.

• The microflora hypothesis of allergy raises the hope of "educating" host immunity, but research into the normal and altered microbiota composition is needed for clinical applications.

## Probiotics and Allergic Disease

### Establishing the Link Between Probiotic Administration and Disease Treatment

Defining the diverse composition of microbiota in human populations is meaningless unless functional links can be made between identifiable microbiota and specific diseases [47]. The pro-biotic concept has a long history, but progress in the scientific and medical evaluation and validation of specific products has been slow [48]. A possible molecular mechanism for tolerance is illustrated by recent studies using high-throughput technologies that suggest the bacterial-host interaction may induce the expansion of T regulatory cells and the expression of immunomodulatory cytokines such as interleukin (IL)-10 and transforming growth factor (TGF-β) [49]. However, these interactions are very complex and involve networks of genes, receptors, signaling molecules, and patterns of disease. Sampling, analysis, and the evaluation of response to intervention are difficult to carry out, and sophisticated bioinformatics and systems biology techniques are needed to interpret the complex interactions and extensive amount of data generated from a large number of subjects [50-52]. Significant data and insights have been provided by metagenome projects undertaken by various centers [31,53]. However, to date, the microbiota associations that have been investigated have been the result of different methods, tools, and host species, and as a consequence the results have often been inconsistent [6,54-57]. Even so, tantalizing outcomes, particularly in studies of eczema in at-risk children, continue to fuel an interest in the field [58,59]. However, a specific stimulus originating from, or the lack of a particular species of, commensal bacteria has not been demonstrated in this setting [60]. Furthermore, these studies have often been misquoted in the review literature [61]. The claim that probiotics work by sustainably altering the intestinal microbiotic niches is not easy to demonstrate from a biological point of view and has not been supported by evidence from human studies. In clinical trials of probiotic supplementation to achieve tolerance, candidate probiotics should ideally achieve a degree of individualization of as-yet unreachable sophistication with respect to the host and its microbiota. The trials should probably also control for additional factors such as diet and vaccination schedules, which will play a role even if the hygiene or microbial origin hypotheses are correct. Thus, the practice of simply flushing the ecosystem with a few chosen species of bacteria without tailoring the probiotics to the host will probably be offset by conserved homoeostatic mechanisms.

Bradford Hill's criteria for causal inference should apply in probiotic research: the strength and consistency of association, temporality, biological gradient, and coherence should be specifically examined [62]. However, relying solely on epidemiological data will not provide all the answers. Although microbiological methods for studying the microbiota have advanced rapidly through the use of high-throughput sequencing, there are still technical challenges that may result in failure to identify whole bacterial communities that have a substantial effect on health, as highlighted by oral microbiologists [63]. However, recent data from the Metagenomics of the Human Intestinal Tract (MetaHIT) project suggest that it may be possible to define a limited number of intestinal symbionts and to carry out investigations to associate them with disease [64]. Generally speaking, the idea that supplemental probiotic bacteria may be used to manipulate evolutionarily conserved homoeostatic mechanisms remains a hypothesis [65].

#### 

##### Take-home message

• The mechanisms by which microbial exposure affects the development and severity of allergic disease needs to be better understood.

• How networks of genes, receptors, and signaling molecules are involved in host-microbiota interaction patterns remains largely unknown. These networks need to be linked to disease states.

• Bioinformatics tools are needed to interpret the relationship of the microbiota compartment with these networks.

• How supplementation sustainably modifies the intestinal ecology remains to be determined for long-term disease outcomes; identifying individuals or populations likely to benefit is not currently feasible.

• More stringent causality assessments should be applied to demonstrate the consistency of an association and biological gradient in the assumed linkage between (undefined) microenvironmental health and allergies.

• Our relationship with our resident microbiota is a highly conserved species-specific trait, unlikely to be amenable to modification by easily manipulated homoeostatic mechanisms.

### Mechanisms of Action

We do not yet understand the mechanisms by which probiotic supplements would be an effective treatment for allergic disease, and the proof of principle for supplementation and reliable clinical use is not clearly established. As this vast area of biomedicine is beyond the scope of the present overview, we focus briefly on 4 areas in need of more basic research before clinical applications may be proposed (the composition of the various microbiota, the gut ecosystem, the pattern of neonatal colonization, and the complex nexus of interrelationships known as commensalism), and within each review the mechanistic rationale for supplementation in the treatment of allergic disease.

#### The Composition of the Microbiota

Microbiologists still argue about the extent of our knowledge regarding what constitutes a "normal" microbiota and how the microbiota may affect the immune system at different stages of development and adulthood [66]. Until recently, most of our microbiological knowledge was derived from cultivable bacteria, and clinical applications were inferred from quantitative analysis of the fecal microbiota [67]. The recent analysis of the microbiota from the fecal samples of a few individuals was made possible by the advent of high-throughput sequencing methods that make use of amplification, cloning, and sequencing of the 16S rRNA genes and revealed a hitherto unseen complexity and biodiversity. One problem with the high-throughput sequencing, however, is represented by technical difficulties involving 'universal" primers and computer software to address a still error-prone technology, which generates a large amount of data [62]. The advent of metagenomics promises an even greater level of power and precision by sequencing the complete genome of all bacteria present in samples taken from the intestinal communities [64].

#### The Gut Ecosystem

The indications for clinical application of probiotics are even less clear than the normal composition of the microbiota. As studies of the gut and other microbiota begin to emerge, it is becoming increasingly clear that closely related species and even strains can interact very differently with their host habitats and various disease states [68]. Studying optimal composition in parallel with clinical applications has never been attempted due to the difficulty of defining both healthy microbiology and of characterizing the fundamental mechanisms underlying perturbations of resident and allochthonous communities (except in some non-IgE-mediated disease states) [54]. Human ecological studies of the biodiversity of the human habitat have revealed, however, that host immune reactions, disease processes, and treatment constitute selective hurdles for supplemented bacteria [48]. We must also think in terms of the effect of supplementation beyond the gut environment. This means factoring in the interrelationships with other bacterial species in flow environments elsewhere in the host. This higher level of complexity contributes to making targeted interventions via microbiota modification even more difficult to plan. After all, we are intervening through a single compartment of the host physiology by supplementing doses of one type of microorganism. To compound the matter, the various habitats within the human host (such as nose, oropharyngeal cavity, trachea, bronchi, and distal genitourinary tract) harbor diverse autochthonous microbiota. These communicate among themselves and with the host through a steady-state outflow of signaling molecules, many fulfilling poorly appreciated immune and metabolic functions for the host, [69] and all contributing to microenvironmental homoeostasis between host and commensal species.

#### Neonatal Colonization

When what is known about neonatal colonization is factored in, more complexity arises. The maternal vaginal and fecal microbiota, as well as breast milk, [70] are the primary sources of bacteria for colonization and succession in the sterile neonatal intestine. Because we do not know what constitutes a "healthy" (infant) microbiota, targeting intervention during a hypothetical "window of opportunity" for improved or therapeutic microbiotal modulation is even more difficult than later treatment. Furthermore, our preliminary investigations have revealed a complex hierarchy of metabolic or immunological possibilities due to many novel bilateral genome adaptations [71]. External (eg, human, animal, and environmental contact; hospitalization; antibiotic use) and dietary (eg, introduction of solids, fermented foods, live yogurts) factors are likely to influence the infant and toddler's highly variable developing gut ecology with transients and relative proportions of a wide range of species [72].

#### Commensalism

The epidemiology of bacterial colonization and succession in the human host remains to be explored, especially because the role of a bacteria-host dialog involving bacteria to-bacteria signaling, bacteria-mucin dynamics, [73] and bacteria-enterocyte crosstalk [55] is increasingly invoked to unravel the cellular and molecular complexity these networks display [53]. Clinical applications are still hampered by our limited understanding of these nested mechanisms.

The appropriate indications, length of treatment, dose, and species to use to modulate the immune system remain to be fully elucidated. Thus, to date, clinicians cannot prescribe with any certainty. Even the selection of desirable bacterial species is empirical. Despite the lack of solid evidence to support their clinical use for long-term protection or immediate treatment, the use of probiotics for at least some patients remains hypothetically valid.

##### Take-home message

• Our knowledge of the composition of the autochthonous microbiota is limited: its variations with development and aging, in health and disease are poorly known.

• The gut ecosystem is only one human habitat and the way it is linked to systemic disease is still to be fully investigated. Interventions designed to make microorganisms work for us should be ecological.

• To take advantage of the "neonatal window of opportunity" for intervention, we would need a better understanding of epigenetics and genomics, both human and microbial.

• We are far from being able to monitor, modulate, or construct an individual treatment rationale predicated on the window of opportunity represented by the early process of microbial colonization.

Three scenarios regarding the fate of probiotic supplements in vivo.

The gut is a river-like ecology: various niches are occupied in a flow dynamic model where microbial signaling, antimicrobial compound warfare, and horizontal gene transfer are in a constant flux. "Flushing" such a stable-unstable microenvironment with billions of colony-forming units of potentially "alien" bacteria mimics the real-life situations of intake of food or other substances. This leads to 3 basic explanations for why--generally--nothing much happens to upset the digestion of the human host:

1. The influx meets the ecological requirements for acceptance by the resident microbiota: the treatment does not achieve its purpose because the immune system does not kick in or is not affected.

2. The influx generates rejection mechanisms from the resident microbiota because all niches are occupied: the treatment does not work because of selection pressure resulting in the probiotic bacteria being "flushed out."

3. An exchange of genes and niches occurs through quorum sensing and chemical warfare for environmental homoeostasis, resulting in a beneficial alteration in immune response. As the default immune response is tolerance, nothing much happens to upset the host daily routine, which may include the continued expression of an allergy phenotype.

The gut is one of the largest immune organs in the body and is the seat of the most active homoeostatic mechanisms known to physiology. Most microbiological events are geared toward the establishment of an equilibrium between resident and allochthonous species and host defenses. These complex biological mechanisms are evolutionarily conserved and are resistant to modification by relative newcomers on the scene, as studies of the microbiota increasingly reveal.

### Quality of Clinical Studies (Evidence-Based Medicine)

Even today, adequate information from which the consumer and health professional can judge the efficacy and safety of retailed probiotics is partial or lacking altogether. More than half of the probiotic papers in the PubMed database are reviews, not reports of the results of experimental or medical science. Additional difficulties in interpreting health benefits associated with probiotics include trials that use experimental preparations instead of actual probiotic products available to the clinician and variable outcomes between results from independent trials.

Several randomized clinical trials examining the role of probiotics in allergic diseases have been published in the past few years, and the quality of clinical studies on probiotics has been assessed in several metaanalyses. In 2007, prevention of allergic disease and/or food hypersensitivity outcomes were assessed by 6 studies reporting a total of 1549 infants [25]. Randomization, allocation concealment, and blinding of treatment were judged adequate, but loss to patient follow-up was considered excessive (17- 61%). Heterogeneity between studies was listed as a bias in a meta-analysis of 5 studies of probiotics administered to reduce eczema in a pooled total of 1477 infants. No benefits were reported for any allergic disease or food hypersensitivity outcome.

Boyle analyzed 12 randomized controlled trials (*N *= 781 children),11 of which tested *Lactobacillus *species, alone or in combination with other probiotics, for the treatment of eczema. The quality of studies varied, and many failed to adequately report the randomization and blinding methods used. Five studies compared probiotics with placebo on their ability to reduce Scoring Atopic Dermatitis (SCORAD) scores, but none yielded significant results. The same lack of evidence was found when quality of life was evaluated in 2 studies. The authors concluded that the heterogeneity between studies may be attributable to probiotic strain-specificeffects [74].

Another meta-analysis evaluating the role of probiotics in the treatment of pediatric eczema reported asignificant difference in SCORAD in the group using probiotics, when compared against the placebo group (see Probiotics and Atopic Eczema). However, the analysis of the duration of treatment, probiotic strains, and the age of patients was not able to identify significant differences between the probiotic and the placebo groups [75].

A further metaanalysis examined the effect of probiotics on prevention and treatment in pediatric eczema. It included 21 trials (*N *= 1898 children) published between February 1997 and May 2007, with 10 double-blind, randomized controlled clinical trials (6 prevention studies [*N *= 1581] and 4 treatment trials [*N *= 299]). These studies supported the use of probiotics in the prevention of pediatric eczema, but the clinical significance of the treatment effect on SCORAD reduction was questionable [24].

#### 

##### Take-home message

• The probiotic literature consists mainly of reviews, few of them systematic. Meta-analyses are rare.

• The results of clinical trials are not reproducible in everyday clinical practice because the probiotics used are exclusive to the research setting.

• Issues that have weakened systematization include compliance, comparator definition, effect of treatment definition, randomization and blinding, probiotic diversity, patient heterogeneity, and lack of evidence.

• The quality of the studies varied, but that meta-analyses were possible is encouraging.

• These biases argue in favor of a "back-to-the-drawing board" recommendation for probiotic research.

## Probiotics and Respiratory Allergy

### Asthma

Experiments on mice have demonstrated that antibiotics, by altering the intestinal microbiota, enhance allergic airway responses and that oral probiotics can modulate allergic responses in the lower respiratory tract [76-78]. However, probiotics as mucosal immune modulators targeting asthma outcomes or parameters have primarily been used for prevention purposes through neonatal supplementation. When administered to adult mice, the supplementation with some probiotic strains may improve asthma features such as airway eosinophilia, local cytokine responses, and bronchial hyperresponsiveness [78,79]. It is against this backdrop that some researchers have explored the potential of probiotic therapy in human asthma.

A trial of a synbiotic preparation in 1223 pregnant women with an increased risk for allergy in their offspring illustrates the difficulties of applying the results of studies from a prevention setting in the context of treatment. The women received a mixture of 4 probiotics for 4 weeks before delivery, and their infant received the same probiotics in combination with a prebiotic for 6 months from birth. The children were then followed up until their fifth year and monitored for allergy development. As a secondary outcome of the study, asthma was observed to develop among 121 children, but no difference in incidence rates was observed between the groups. However, the conclusions of this trial appear relevant for prevention and not treatment purposes, as the main difference was lower rates of allergy among infants born by caesarean section [80].

In a randomized trial, 231 newborns (though not their mothers) received *Lactobacillus acidophilus *supplementation for 6 months. This did not protect them against wheezing during their first year of life [81].

In another prevention study, the supplementation of infants at risk of allergy with *Lactobacillus reuterii *for 1 year failed to influence asthma prevalence rates at 2 years. In this study carried out in an eczema prevention setting, respiratory allergy was only a secondary outcome measure [82].

In a rare study reporting negative outcomes from probiotic use, prescribing *Lactobacillus rhamnosus GG *supplements for 6 months to 105 infants in a primary allergy prevention setting actually increased the prevalence rate of recurrent (≥ 5) episodes of wheezing bronchitis among patients treated with probiotics, but only 17 infants had developed wheezing and the outcome was not primary [83].

In a randomized double-blind trial, an unselected population of pregnant women was treated with *L. rhamnosus GG, L. acidophilus *La-5, and *Bifidobacterium animalis *subsp. *lactis *Bb-12 from the 36th week of gestation until 3 months postpartum while breastfeeding. The primary endpoint was atopic disease. Asthma was monitored separately during the first 2 years. However, no significant effect of the intervention emerged during this period [84].

Thus, although inhalant allergen sensitization appeared slightly downmodulated in subgroup analyses of 2 populations, [83,85] neonatal studies do not associate probiotic treatment with reduced prevalence of inhalant allergen sensitization [85].

Supplementation is not without its risks. There is a documented association between *L. rhamnosus GG *administration and cat allergen sensitization [84]. The effects on allergic airway symptoms are mixed. Reports of an increased proportion of children with frequent wheeze during the first 2 years of life after perinatal administration of *L. rhamnosus GG *have begun to appear, [85] similar to findings in a mouse model of *Lactobacillus casei *intervention [86].

Clinical treatment trials of probiotics specifically targeting asthma are rare, and relatively short follow-up periods (usually 1-2 years, sometimes 4-5 years) are a feature of these studies.

In one study of preschoolers, 187 children aged 2 to 5 years were given fermented milk containing 10^10 ^cfu *L. casei*, or placebo, each day for 12 months. There were no statistically significant differences between the groups in terms of cumulative incidence of asthma episodes [87].

Two secondary prevention studies have recently been published. In the first study, 71 infants with atopic eczema (median age 5 months) were given supplements containing a synbiotic mixture of *Bifidobacterium breve *M-16V and a proprietary fructo- and galactooligosaccharide mix or a placebo for 3 months. At the 1-year follow-up, the prevalence of frequent wheezing and wheezing and/or noisy breathing apart from colds was significantly lower in the group receiving the synbiotic (13.9% vs 34.2%, with an absolute risk reduction of 20.3% in favor of the synbiotic). However, these were secondary findings in a study targeting patients with eczema for their respiratory symptoms [88].

In the second study of infants at risk of allergic disease, *L. rhamnosus GG *10 was given for 6 months to 131 subjects aged 6 to 24 months presenting with ≥ 2 episodes of wheeze and a family history of atopic disease. Asthma-related events (need for inhaled medicine, symptom-free days) and atopic eczema events were recorded for a 1-year period, during which no difference in asthma-related events reached statistical significance [89].

Among older children and in adolescence or young adulthood, the efficacy of probiotic supplementation for respiratory treatment appears to be limited for methodological considerations.

In a study targeting 6- to 12-year-old children with asthma and allergic rhinitis, *Lactobacillus gasseri *A5 supplements were given for 2 months. Pulmonary function and peak expiratory flow rate increased significantly and clinical symptom scores relating to asthma and rhinitis decreased in the group treated with the probiotic. This study was, however, limited by its short study duration and small sample size [90].

Twenty-nine adult asthmatics with house dust mite allergy were randomized in a double-blind study to receive a synbiotic (*B. breve *M-16V with galacto- and fructooligosaccharide) during a single month. The primary outcome was allergen-induced bronchial inflammation. A secondary outcome, peak expiratory flow, improved significantly in the group receiving the synbiotic [91].

In a population of 18 teenagers and young adults who presented with allergic rhinitis, 7 patients with asthma were randomly treated with *L. rhamnosus GG *or placebo for a total of 4.5 months before, during, and after the birch pollen season. There were no significant differences between the prevalence of nose and eye symptoms. The cumulative use of allergy and asthma drugs increased in the probiotic group and, compared with baseline, no significant differences were found during the pollen season. Overall, supplementation did not improve respiratory symptom scores [92].

In a 1997 double-blind crossover trial, 15 adults with moderate asthma were given yogurt with or without *L. acidophilus *in 2 single-month treatment phases. Similar to the study with *L. rhamnosus GG *described above, this study was not able to match a net benefit of supplementation in terms of immune parameters (a modest improvement of eosinophilia and increased interferon gamma expression) with significant differences in peak expiratory flow or spirometry assessment [93].

#### 

##### Take-home message

• Proof of principle for probiotic use in asthma has been inferred from murine models.

• What we know about probiotics in asthma is derived from prevention studies.

• No primary prevention study has been able to demonstrate an effect of probiotic supplementation in asthma in humans [94].

• The quality and power of some studies have been questioned [82].

• There is as yet no evidence that probiotic supplementation modulates disease phenotype: supplementation is not therapy.

• Positive studies should be replicated in larger samples and for a longer period of time.

• Adolescents and young adults may not provide the best setting for intention-to-treat studies because preexisting allergen sensitization, atopy phenotype, and stage of allergic disease may confound treatment efficacy.

### Allergic Rhinitis

Epidemiologic evidence indicates that immune responses in the gut may modulate responses in distant target organs, including the nose, [46] and immune effects beyond the gastrointestinal tract have been documented [95]. Probiotics have been shown to alleviate symptoms and to affect markers of allergic inflammation, including a decrease of eosinophil infiltration into the nasal mucosa [96]; decreasing IL-5 production [97]; and increasing TNF-α, interferon (IFN)-γ, IL-10, IL-12, and IL-13 production in adults and children suffering from pollen or dust mite allergy. These considerations form the tenuous background on which probiotics, including the *Bifidobacterium longum BB536, L. casei *Shirota, *Lactobacillus paracasei *LP33, *L. acidophilus *L92, and *L. paracasei ST11 *strains have been proposed for treatment of allergic rhinitis [98-103]. It is noteworthy that these data were obtained in experimental settings and that, yet again, the heterogeneity of reporting precludes meta-analyses.

### Perennial Allergic Rhinitis

In children, the use of fermented milk fortified with *L. paracasei *LP33 has been proposed for the treatment of perennial allergic rhinitis (PAR) and has achieved significant reduction in pediatric rhinitis quality of life [104]. In a study of preschoolers treated with milk fermented with 10^10 ^cfu *L. casei *or placebo for 12 months, a difference in the cumulative incidence of rhinitis episodes was found [89]. In another study, a small number of 6-to 12-year-old children who suffered from asthma and allergic rhinitis were given *L. gasseri *A5 supplements or placebo. The rhinitis symptom score decreased in the treated group [90]. Finally, a particular form of PAR was assessed in nonprofessional marathon runners but *L. rhamnosus GG *supplementation did not yield a positive effect [104].

### Seasonal Allergic Rhinitis

Seasonal allergic rhinitis (SAR) may be triggered by the pollens of numerous plant families, and studies of the effect of probiotics on SAR have been performed in a series of models. In a Finnish study testing *L. rhamnosus *GG versus placebo for the treatment of birch pollen-induced allergic rhinitis, no significant differences in rhinitis symptom scores were found [92]. In another Finnish study, 47 children with birch pollen allergy randomly treated with a combination of *L. acidophilus *NCFM and *Bifidobacterium lactis *ATCC SD5219 or placebo experienced a nonsignificant reduction in nasal symptoms. This specific combination of probiotics reduced pollen-induced infiltration of eosinophils into the nasal mucosa [96]. A UK study found that *L. casei *Shirota, administered via a milk drink for 5 months, significantly reduced the levels of antigen-dependent IL-5, IL-6, and IFN-γ in grass pollen-induced rhinitis. Levels of specific IgG increased and specific IgE decreased in the probiotic group [103]. A fourth study, testing an ad hoc mixture of *L. rhamnosus *GR-1 and *Bifidobacterium adolescentis *supplied in a yogurt, found no clinical effect in a group of adult patients with ragweed-induced allergic rhinitis. However, potential desirable effects were found in the cytokine profiles of these patients, albeit without a clinical symptom tie-in [105].

Several studies have focused on Japanese cedar pollen (JCP)-induced allergic rhinitis. Two studies of intervention with *Lactobacillus plantarum *14 (LP14) in female students found a significant improvement in ocular symptom-medication score that was associated with the inhibition of postexposure eosinophil counts [106]. A randomized, double-blind placebo-controlled trial of 44 subjects allergic to Japanese cedar and treated with *B. longum *BB536 for 13 weeks during the pollen season found significant decreases in clinical scores in the treated group. These results were attributed to immunomodulation of a T-helper 2 (Th2)-mediated immune response [102] and to a "stabilization" of fluctuations in the composition of the patients' fecal microbiota [107,108]. In contrast, in this same group, numbers of *Bacteroides fragilis *and *Bacteroides intestinalis *were significantly higher among JCP-allergic patients and correlated positively with symptom scores and JCP-specific IgE levels [109].

Another double-blind placebo-controlled study examined the effect of *Lactobacillus *GG and *L. gasseri *TMC0356 in the same setting. A yogurt prepared with these bacteria and administered to 40 subjects with a clinical history of JCP-allergic disease significantly decreased the mean rhinitis symptom score. These effects were attributed to a specific downregulation of the Th2 immune response [99]. The results from this study suggested that stabilization of the intestinal microbiota by the selected probiotic strains was associated with these clinical findings [110] and that the diversity of intestinal bifidobacteria could be a prospective target for using probiotics in the management of JCP [111].

Intriguingly, a recent study showed that recombinant lactic acid bacteria expressing a major JCP allergen had both allergen-specific and non-allergen-specific clinical effectiveness. These bacteria suppressed the allergen-specific IgE response and nasal symptoms in a murine model of cedar pollinosis [112].

Despite these enthusiastic reports, it should be noted that in many studies the primary clinical outcome was negative, and the quality of design has been judged as poor. A specific mechanism linking intestinal effects with an antiinflammatory or immunosuppressive effect on the local upper respiratory tract has yet to be defined, and such a mechanism is needed to provide us with the grounds for their clinical use. Unlike in other clinical fields, in rhinitis, the effect of probiotics has primarily been demonstrated for treatment, whereas their benefits for prevention remain inconclusive.

#### 

##### Take-home message

• The clinical use of probiotics in allergic rhinitis are based on postulated effects beyond the gastrointestinal tract.

• The literature suggests that the disease may be subdivided into several phenotypes (PAR, SAR, JCP induced). This can be misleading in investigating treatment effects in pollinosis.

• The heterogeneity of studies precludes meta-analysis.

## Probiotics and Food Allergy

Very few studies have explored probiotic therapy for food allergy, and no systematic review seems possible currently. Furthermore, the clinical information regarding probiotics and food allergy is derived from studies of patients with eczema who may represent a specific phenotype of disease. Proof of principle in humans is thus lacking from population studies. A narrative summary of findings from selected studies follows, but no recommendation for the use of probiotic therapy in this clinical setting may currently be extrapolated.

### Studies reporting beneficial therapeutic effects

In 1997, a group of infants with cow's milk allergy, atopic eczema treated topically, and a positive family history of food allergy were randomized to receive an extensively hydrolyzed formula with or without *Lactobacillus GG *to alleviate their skin and food allergy symptoms. The primary objective was to reduce inflammation in the setting of a cow's milk elimination diet. A single month of treatment with the probiotic was followed by 1 month with the hydrolysate. The patients were then reexamined and orally challenged with cow's milk. The atopic eczema score markedly improved in the active treatment arm of the study (from 26 down to 15), but not among untreated patients. It also improved (from 26 to 11) in a group of infants who were breastfed while their mothers received *Lactobacillus GG *for 1 month. However, there was no effect of supplementation on cow's milk allergy symptoms [113].

In a subsequent study, the same researchers fed an extensively hydrolyzed formula with or without *Lactobacillus GG *and *Bifidobacterius lactis Bb-12 *to infants who had developed atopic eczema although exclusively breastfed. There was a significant improvement in atopic eczema in the 9 infants who had received probiotic supplementation, but only in 4 of 9 controls [114]. Again, no effect was reported on food allergy. A similar lack of effect of probiotic supplementation was noted using a combination of *L. rhamnosus *and *L. reuteri *[115]. However, another study reported a beneficial effect of *L. rhamnosus GG *but not of a mixture of *B. breve, Propionibacterium freudenreichii*, and *Propionibacterium IS *[116].

What emerges from these studies is that probiotic supplements are reported to be effective in the prevention of eczema while proof of clinical efficacy in food allergy is still lacking.

### Studies reporting no beneficial therapeutic effects

A study of infants with atopic eczema concluded that there was no benefit of adding *L. rhamnosus *or *L. rhamnosus GG *to an extensively hydrolyzed formula. Neither was there a significant difference in allergy (total and specific IgE), inflammatory markers, or cytokine production by peripheral blood mononuclear cells after supplementation [117]. Another study showed no therapeutic advantage of *L. rhamnosus GG *over placebo for atopic eczema in infants with food allergy [118]. In a group of children with eczema younger than 10 years of age, the daily intake of a mixture of *L. rhamnosus *and *B. lactis *for 12 weeks did not differ from placebo in improving atopic dermatitis [119]. Nevertheless, a significantly greater improvement was noted in food-sensitized compared with non-food-sensitized patients, but the observed improvement was not sustained 4 weeks after cessation of probiotics. Similarly, young infants with milk allergy treated with an extensively hydrolyzed formula supplemented with placebo or with *L. casei *and *B. lactis *did not display differences in tolerance to cow's milk at 6 and 12 months [120].

### Studies reporting no beneficial preventive effects

In an Australian study, 218 infants whose mothers were atopic were randomized to receive, from birth until 6 months of age, either a placebo or 3 × 10^9 ^cfu *L. acidophilus *daily. By 6 months and 12 months of age, the incidence of atopic eczema in the *Lactobacillus *group was similar to that in the placebo group. Surprisingly, the probiotic group had a higher frequency of positive skin tests to common foods and aeroallergens than the placebo group (40% vs 24%) [81]. A Finnish study reported the effects of administering, for 2 to 4 weeks before delivery, a mixture of 4 probiotics (two *Lactobacillus rhamnosus *strains, *Bifidobacterium *spp, and *Propionibacterium *spp) to women pregnant with infants at high risk of atopy. The infants were then given a placebo or the probiotic mixture plus a prebiotic for 6 months [121]. By 2 years of age, the 2 groups showed no significant difference in the cumulative incidence of any allergic disease or sensitization to food allergens. Another Australian study found a similar lack of treatment effect in the rate of development of allergy or of IgE sensitization by 5 years of age in children whose mothers had received a probiotic mixture during the last month of pregnancy and who had received the same probiotics for the first 6 months of life [83]. Probiotic intake was associated with a significant reduction in allergy incidence (24.3% vs 40.5% in those who received placebo) when birth by caesarean section was considered [80]. Similar findings have been reported by others [85,122]. A study of 3 European birth cohorts has shown no relationship between the development of atopic eczema or food sensitization [60] and the type of intestinal commensal bacteria; this is in contrast to most observational studies of intestinal microbiota composition and eczema/atopy in infancy [60].

The Diagnosis and Rationale for Action Against Cow's Milk Allergy [123] and National Institute of Allergy and Infectious Diseases (NIAID) Guidelines [124] do not recommend the use of probiotics for milk or food allergy. However, the NIAID Guidelines suggest that in the prenatal and early neonatal periods, probiotics may be associated with a slight reduction in the incidence of eczema. The most significant results were seen when probiotics were used in conjunction with breastfeeding or a hypoallergenic formula. So far, studies have yielded inconsistent findings on the usefulness of probiotics in food allergy. The discrepancies may be attributed to variation among studies in multiple factors related to the probiotics (type, dose, mixtures, and duration) or to the recipient (birth method, type of feeding, and age). The available findings suggest that there is greater potential for prevention than for therapy. More studies are needed to delineate the role of probiotics in allergy practice [125].

#### 

##### Take-home message

• The lack of effect of probiotic treatment in the setting of food allergy is not related to the lack of evidence of its effect in the general context of prevention through immunomodulation.

• Probiotic treatment studies have suffered (being dietary interventions) from coadministration with adjuvant therapy (prebiotics or synbiotics)

• Failing to make a distinction between maternal treatment and fetal/neonatal treatment in intervention studies has reduced the generalization of findings

• It is likely that food allergy treatment cannot be entirely dependent on environmental or dietary factors.

## Probiotics and Atopic Eczema

There is a considerable body of literature published to date, with at least 14 randomized controlled trials for treating eczema with probiotics and 14 for preventing eczema in humans. Unfortunately, these studies are often misquoted, [67] and attempts at systematization have been thwarted by the heterogeneity of probiotic products, study protocols, and allergy markers used in analyses of the immune modulation induced by probiotics. The issue of which probiotic is used is inconsequential. It has been known for some time that individual strains pulse dendritic cell activation differently. Thus, the cytokine profile emerging in response to treatment is substantially altered, thereby confounding clinical interpretation [126]. Critical to the endorsement of treatment with supplemented probiotics are 2 of the most quoted studies in the prevention literature, from which much of the rationale for treatment is derived. Both studies used the same microorganism and similar protocols, but achieved diametrically opposite eczema outcomes: one claimed to show efficacy, whereas the other showed a lack of clinical effects [20,88].

In the treatment of eczema, most studies have tested *Lactobacillus *species, either alone or in combination with other probiotics and/or prebiotics. One proof-of-principle study specifically targeted adults [127]. Systematic review and/or meta-analysis of 12 of the 14 published studies have been undertaken by 3 separate groups [see Quality of Clinical Studies (Evidence-Based Medicine)]. A Cochrane systematic review and one other meta-analysis found no evidence that probiotics are effective for treating eczema, albeit with significant heterogeneity between the outcomes of different studies [25,26]. A third meta-analysis found a statistically significant effect of probiotic treatment on the mean change in SCORAD index from baseline to the end of treatment [mean difference between groups -3.01 points; 95% confidence interval (CI), -5.36 to -0.66; *P *= 0.01]; however, this effect size is of limited clinical significance [81].

All but one preventive study evaluated lactobacilli (7 used *Lactobacillus rhamnosus *strain *GG*), either alone or in combination with other probiotics and/or prebiotics [64,65,89-91,114,120,128-134]. At least 10 studies limited recruitment to women carrying a fetus at high inherited risk of allergic disease. However, the studies were heterogeneous in other respects, particularly in the timing of the intervention. In 10 studies, treatment was started during pregnancy, in one during the first trimester, and in the others in the last trimester. In 3 of the last trimester interventions, maternal treatment stopped with delivery, and in 7 it was continued for some time during breastfeeding. In 10 studies, a probiotic was given directly to all infants during the postnatal period, usually commencing early, but in 3 studies administration started after 3 months. Treatment ceased within the first year in almost all studies, except for one study, which continued for 2 years. No study replicated the design and intervention of a previous successful prevention trial, with the exception of the study by Kopp and colleagues, which yielded different findings to the similarly designed study by Kalliomaki et al [64,90] With these caveats in mind, a number of authors have undertaken systematic reviews and/or meta-analyses of the randomized controlled trials evaluating the probiotic effect in eczema prevention. Two meta-analyses have found no evidence that probiotics prevent the development of atopy. A Cochrane systematic review included 5 of the 14 trials in meta-analysis, finding a relative risk of 0.82 (95% CI, 0.70-0.95) attributable to probiotic treatment [26]. A more recent meta-analysis that included 12 of the 14 trials similarly found a relative risk of 0.79 (95% CI, 0.67-0.92) [24]. Neither metaanalysis found evidence that probiotics can prevent the development of atopy. There was significant heterogeneity in study outcomes in these meta-analyses (I^2^, 64 and 31%). Subgroup analysis suggested that infant treatment without maternal treatment may be ineffective [24]. The authors of the Cochrane review advised caution in interpreting the results of their meta-analysis, due to heterogeneity and high rates of loss to follow-up (17-61%) in the individual trials [26].

### 

#### 

##### Take-home message

• There is currently no meta-analytic evidence that probiotics are clinically effective for treating established eczema.

• The possibility that novel probiotic strains or treatment during adulthood may prove effective cannot be discounted.

• Further research into probiotic design and/or patient indication is warranted.

• Drawing inferences for therapy from prevention studies may be misleading.

• Meta-analytic evidence suggests probiotics may be effective for preventing eczema, but due to heterogeneity in study designs and outcomes it is impossible to issue clear recommendations at present.

• Further basic research into probiotics should address the question of the optimal mode of administration.

• The lack of clear mechanisms of action (intestinal barrier function and systemic immunity in particular) in the context of the developing infant intestine prevents optimizing interventions [135].

• It is unclear how probiotics exert their effect on the various pathogenetic aspect(s) of eczema, if at all.

## Probiotics and allergic gastrointestinal disease

Gastrointestinal food allergy may present as a range of clinical syndromes in infants and young children. The 3 main gastrointestinal allergic manifestations are food protein-induced proctocolitis, food protein-induced enteropathy, and food protein-induced enterocolitis syndrome [118]. Eosinophilic esophagitis has recently been identified as another condition closely related to food allergy, particularly in children [136]. Symptoms of gastrointestinal food allergy include persistent diarrhea, rectal bleeding, vomiting, failure to thrive, irritable behavior, and feeding difficulties [137,138]. These conditions are caused by non-IgE-mediated mechanisms and are generally characterized by noninfective gastrointestinal inflammation with an increase in mucosal T lymphocytes, eosinophils, mast cells, or basophils. However, these allergic manifestations provide a paradigm of how interventions may be designed and carried out.

Probiotic effects are thought to depend on innate mechanisms of immunity via Toll-like receptors, which promote Th1 cell differentiation; the production of regulatory cytokines (IL-10 and TGF-β); and an enhanced intestinal IgA response [139,140]. The use of probiotics for the treatment or prevention of gastrointestinal conditions therefore appears plausible, although no direct supportive evidence regarding gastrointestinal food allergy or eosinophilic esophagitis is available at present. Importantly, animal models on the effects of various lactobacilli and bifidobacteria suggest that there may be significant strain-specific differences as to how probiotics affect innate and adaptive immune responses [141].

The integrity of the epithelial barrier function in the gut is a key factor in preventing gastrointestinal inflammation and maintaining gut health. Impaired barrier function may lead to inflammatory gastrointestinal conditions, including inflammatory bowel disease or celiac disease, and delay the recovery from infective gastroenteritis [142]. Probiotic treatment has been advocated to improve or maintain barrier function by regulating epithelial tight junctions [143]. However, research has yet to ascertain whether these effects result directly from the probiotic used or from increased selective pressure among the resident microbiota. Although, in theory, probiotic bacteria could reduce the risk of food protein-induced gastrointestinal manifestations, this has never been demonstrated in clinical trials.

Probiotic treatment has been attempted for a range of gastrointestinal conditions, including viral gastroenteritis, [144] infantile colic, [145] and necrotizing enterocolitis of premature infants, [146] with mixed results. However, the potential for these interventions has not been prospectively studied in humans.

In summary, there are to date no data specifically assessing the effects of probiotics on gastrointestinal food allergy [119,147]. Randomized clinical trials are therefore required before the use of specific probiotic strains may be recommended for the prevention or treatment of gastrointestinal food allergy.

### 

#### 

##### Take-home message

• Currently there are no data specifically assessing the effects of probiotics on gastrointestinal food allergy.

• Randomized clinical trials are required in the setting of gastrointestinal non-IgE-mediated food allergy.

## Some safety aspects of probiotics for allergy treatment

A major theoretical risk from the use of probiotics in the treatment of allergy is that the documented benefits in a small number of allergic conditions will not be borne out by strong evidence of efficacy. Their wide use is due, in part, to the belief that a bewildering range of allergic symptoms and conditions may be improved through probiotic consumption. However, problems linked to the clinical use of novel probiotics without a long history of safe use are bound to occur when products derived from nonhuman strains or from the gut microbiota of healthy humans are used in a community setting.

Species in *Lactobacillus *and *Bifidobacterium*, the genera to which most probiotic supplements belong, are generally considered to be safe as food additives [148-150]. *Lactobacillus *species, however, have been identified as cariogenic [151-153]. Lactobacillemia (or *Lactobacillus *bacteremia) is a recognized entity that has been implicated in infective endocarditis [154,155] and the aggregative potential of the genus has been characterized [156]. Experimentally, the intraperitoneal injection of group B *L. casei *cell walls produces an inflammatory coronary arteritis in mice that mimics the arterial damage found in children with Kawasaki disease [157]. Dietary supplements are not regulated for purity and potency, as pharmaceuticals or biological agents are, and thus the various regimens in which probiotics are used in allergy treatment have not been tested in exactly the same conditions. Adverse symptoms or severe systemic disease cannot be correlated in the absence of extensive preclinical studies. Nowhere is this lack more acutely felt than in the case of enteral feeding formula, where genera containing many pathogenic species and strains are used without excluding the possibility that during mixing and preparation harmful interactions could occur between the probiotic and the formula [158].

The criteria for probiotic product safety are industry standards, but problems remain, and one national survey documented the presence of undeclared species or taxonomically incorrect or fictitious microbial names in 51% of the products it sampled. One preparation contained numerous spores with a content of 4.5 × 10^6 ^cfu/g *Bacillus cereus *and 3 others had 8 × 10^5 ^to 6 × 10^6 ^cfu/g *Bacillus subtilis *spores [159].

The only common side effects associated with probiotic supplementation are transient gastrointestinal symptoms (such as nonfunctional bloating and flatulence) attributed to a "die-off" effect [160] of bacterial interference during prophylactic treatment [161]. This illustrates an underlying concern of probiotic research that supplementation with allochthonous microorganisms may cause the adverse effects they are supposed to fight, given the unknowns of therapy aimed at immunomodulation [162].

The very properties for which probiotics have been proposed for therapy in clinical practice, however, could easily become a double-edged sword in the allergist's armamentarium. Skewing the Th1/Th2 balance toward Th1 may not be always safe, even in healthy individuals. In pregnancy, for instance, a bias toward the Th2 phenotype is held to be important to maternal-fetal immune tolerance [163]. Stimulation of Th1 immunity would also not be recommended in patients with autoimmune diseases that are mediated by a Th1 cytokine profile, especially early in the course of disease [164].

In immunocompromised hosts, in addition, lactic acid bacteria have been involved in human disease. This assumes importance in the context of widespread administration in neonatal cohorts, as in the case when probiotics are included in starting formulae.

Safety concerns, therefore, have always been part of probiotic research, and are carefully considered by the industry during the process of probiotic bacterial strain selection [165]. Despite these hypothetical caveats, however, the safety of probiotic products in clinical practice remains limited by the absence of clinical trial evidence rather than by demonstration. This may be attributed to an overestimation of efficacy outside research settings or to the lack of well-designed clinical trials reporting on safety considerations [166].

Even the immune safety of probiotics should not be taken for granted, as reports of sensitization and anaphylaxis to probiotic supplements have begun to appear, and 3 articles have reported a total of 99 cases [167-169].

In the course of investigating the immunomodulatory effects of probiotic bacterial strains, researchers found a novel bacterial strain that suppressed the induction of oral tolerance. Although *Lactococcus lactis *BB356 increased antigen-specific IFN-γ production, it decreased antigen-specific IL-4 and IL-10 in rodents [170].

In conclusion, the theoretical risks outlined here are all contingent on the yet-to-be demonstrated consistent systemic influence of supplements to harness the power of the healthy microflora. Similarly, emphasizing the inevitable downside of biotherapeuticals in widespread use can only add evidence of indications where they may be safely and reliably used [97].

### 

#### 

##### Take-home message

• Lactobacilli and bifidobacteria are generally considered safe.

• Episodically, concerns have been expressed over the possible cariogenicity of lactobacilli and the contribution of *Lactobacillus *to bacteremia in infective endocarditis and experimental Kawasaki disease.

• Safety issues are of utmost importance when probiotics are added to cow's milk formulae intended for general use.

• Immunocompromised hosts can be subject to probiotic-induced bacteremia.

## Structure and correspondence of claims with the state of the art in allergy research

Regulators have attempted to clarify for the consumer the possible antiallergic properties of probiotics. To fill an earlier vacuum, an international consensus on methodology to assess the efficiency and safety of probiotics was issued by both the United Nations Food and Agriculture Organization (FAO) and the WHO. These "Guidelines for the Evaluation of Probiotics in Food" concluded that: "the health benefits for which probiotics can be applied include conditions such as ... allergy."[22] However, the sentence was deleted in the FAO/WHO 2002 guidelines [171]. The recommendations as set out in the later report formed the basis for further regulatory actions by the US Food and Drug Administration and, lately, by the European Food Safety Administration (EFSA). In 2010, EFSA published a "Scientific Opinion on the Substantiation of Health Claims Related to Various Food(s)/Food Constituent (s) Claiming 'Healthy and Balanced Digestive System', Increasing Numbers of Gastro-Intestinal Microorganisms and Decreasing Potentially Pathogenic Gastrointestinal Microorganisms Pursuant to Article 13 of Regulation (EC) No 1924/20061."[172] Allergy was not even an issue examined.

In the 10 years since the publication of the WHO guidelines, their recommendations have seldom been quoted. In particular, the criteria for claims that remain outstanding are rarely the objects of probiotic literature: safety, structure, and function of "allergy improvement' claims; generic and product-specific claims; and product efficacy and effectiveness remain poorly investigated areas. Claims made in probiotic research are not supported by an evidence-based medicine approach. The measurement of, and criteria for, health benefits and the biomarkers to be selected are not yet universally accepted. General criteria, surrogate endpoints, biomarkers of efficacy in allergy treatment, research needs, and validation of studies are in need of further investigation from a methodological point of view.

## Suggestions for future studies

The balance of evidence from the present overview suggests that probiotic supplements do not have a promising future for the prevention or treatment of eczema, allergic rhinitis, or food allergy. Extensive further research in several fields of biology, microbiology, immunology, allergy, and related fields is needed to clarify these issues.

Current research suggests that the future lies in a multidisciplinary approach to basic research and clinical disciplines in a metascience that does not yet exist. Although the claim that we are able to tailor interventions to modulate the immunobiology of human allergic disease through probiotic supplementation currently belongs to empiricism, nonetheless it has made it into scientific journals; hence the need for this overview.

Too many unknowns remain even to decide whether immune parameters defining phenotypic features for some patients may be improved by supplementation. First of all, there is a need to understand the influence of diet and other environmental factors on the microbiota of pregnant or lactating mothers and their infants through observational studies of populations with different dietary and environmental exposures. We need to identify causal pathways and biomarkers (ie, cytokines and their associated phenotypes) for the health benefits of probiotics in relation to allergic disease. The first steps in this direction may be to understand whether probiotics for eczema prevention act through modulation of breast milk immune modulators (antibodies, cytokines, and growth factors) or microbial composition, through the induction of low-grade inflammation when directly administered to infants, through effects on infant intestinal epithelial barrier function, or via other mechanisms. Effort should be made to define the populations of subjects who benefit most from these interventions. Animal models may provide clues to mechanisms of tolerance. However, recent data demonstrate that colonic bacteria resident in other mammals differ from those typically found in humans. Therefore, caution should be exercised in predicting results in humans on the basis of animal testing. Among other critical research issues, the following appear to be topical.

### Basic Probiotic Science

1. Are behavioral changes related to hygiene and avoidance of microbes (birthing and nursing conditions, water and food sanitation, and cleaning practices) responsible for changes in the establishment of microbial symbioses that reduce tolerance?

2. What is "dysbiosis" and how does it relate to clinical phenotype? Population and genetic studies are needed to answer this question.

3. What is the role of antibiotics, and physicians' antibiotic prescribing behavior, in the current spate of allergic manifestations attributed to "dysbiosis"?

4. What is the composition of the human microbiota in various environments throughout life, in health and disease, and particularly in allergic manifestations? Epidemiological studies are also needed before we can reliably modify the human microbiota for therapy or prevention.

5. What microbial species and strains are tolerogenic or induce regulatory immune factors? How does this vary between subjects in the population?

6. What organs, tissues (in the host compartment), niches, and populations of microorganisms (in the microbiota compartment) are involved in the induction of tolerance: is it the oral cavity, the small intestine, or the colon? At finer anatomy, is it the epithelium, lymphatic organs, dendritic cells, mast cells, B cells, macrophages, T cells, or others that is the primary site from which this activity originates?

7. Is the same site and mechanism as important for early onset of tolerance as for late onset of tolerance or for maintenance of tolerance?

8. What is the link between bacteria, the gut and lung mucosa, the immune system, and the allergic disease states mediated by mucosal bacterial species?

### Probiotic Products for Allergy Treatment

9. Could it be that we do not have sufficient probiotic strains? Do we need to focus on certain species, such as *L. rhamnosus*, which has produced tantalizing outcomes with regard to eczema prevention? Or should there be an effort to identify other microorganisms? Should we try commensals or newcomers?

10. What clinical evidence or markers of disease do we need to establish to determine whether a well-defined probiotic strain is sufficient to modulate host and/or microbial homoeostasis and to do so efficiently? Tolerance is our immunological "default mode," but what cytokine profile or signaling format do we set to define the success or failure of an intervention?

11. Do probiotics provide a necessary or sufficient condition for the establishment of tolerance to antigens? Does the prevention of allergy through tolerance or treatment depend on the concurrent administration of probiotic and antigen?

### Methodological Issues

12. Why have well-designed and sufficiently powered longitudinal, multicenter, birth cohort studies and long-term follow-up studies of randomized placebo-controlled trials failed to convince their skeptics?

13. Basic research into commensals at the microbiota-human interface is needed to test the hypothesis that dietary manipulation via supplementation can affect host homoeostasis, metagenome interchanges, and modulation, together with their downstream effects. This is a whole new field of enquiry and will require many years of multidisciplinary research leading to meta-analyses to identify and validate clinical applications.

14. From a methodological point of view, research should prioritize long-term, sufficiently powered, well-controlled, randomized, multicenter clinical trials in selected and unselected human subjects recruited according to current definitions. Researchers should adopt research standards recommended by learned societies in their field to facilitate future meta-analyses by generating homogeneous databases.

15. Translational research synthesizing the in vitro and ex vivo literature is needed before probiotic supplements may become an independent class of bacteriotherapeuticals.

16. From a regulatory point of view, the transition from functional food supplement to mucosal immune modulator targeting allergy outcomes should follow established practices and should be submitted to scientific society and government agency oversight to end the "probiotic privilege" before claims can be researched in the context of allergy outcomes.

### Clinical Issues

17. Clinical researchers have emphasized the inconsistencies of many probiotic studies in the treatment of allergy, and clinical practice studies have identified the following as the main confounders of this field and, therefore, possible areas of focus for research [173]: defined species and specific strain of probiotic, the dose used, the use of combination versus single type, the method of administration, the age at administration for prevention, the time after disease onset for treatment, the duration of therapy, the coexistence of food allergy, dietary elimination issues, and the limiting of probiotic feeding to a hypoallergenic formula.

18. The potential side effects of therapy in various allergy settings should also be the focus of research.

## The final take-home messages

In the consensus opinion of the panel members of the Special Committee on Food Allergy, the full implications of probiotic supplementation for the treatment of allergic disease remain to be worked out; clearly, probiotics are not for everyone. Even if the hygiene hypothesis and the "microflora" hypothesis of the origin of allergic disease are eventually proved through biological breakthroughs, it remains to be demonstrated whether we may stably modulate the host compartment of the commensal relationship with our resident microbiota. This needs to be demonstrated at the microbiological, immunological, and genetic levels for bacteriotherapeuticals to be accepted by allergists and clinical immunologists in their daily practice.

This is not to say that the "probiotic hypothesis" for allergy treatment is a dead end. On the contrary, there is tantalizing evidence in vitro and in animal models that the future lies in this direction, that is, that one day we will be able to orchestrate research efforts toward making probiotics the agents or adjuvants we need to harness the power of the microbiota to shift for designated patient profiles.

So far, there is no consensus on compartmental dysbiosis and homoeostasis, and thus the jury is still out on whether supplementation is the best intervention to achieve this research objective. A leitmotiv of probiotic research is to present immune modulation by means of probiotics as a fait accompli. We contend just as empirically that, given the weakness of the clinical evidence, probiotic dietary supplements may not be every patient's cup of tea.

## Abbreviations

CUPPA: Clinical Use of Probiotics for Pediatric Allergy; SPT: Skin Prick Test; SAR: Seasonal Allergic Rhinitis; PAR: Perennial Allergic Rhinitis; SCORAD: Scoring Atopic Dermatitis; NIAID: National Institute of Allergy and Infectious Diseases; TGF-β: Transforming growth factor β; JCP: Japanese cedar pollen [-induced allergic rhinitis]; FAO: Food and Agriculture Organization; WHO: World Health Organization; EFSA: European Food Safety Administration; IL: Interleukin; IFN: Interferon; EBM: Evidence-Based Medicine.
